# Bursting through the cell cycle

**DOI:** 10.7554/eLife.14953

**Published:** 2016-03-07

**Authors:** Shani Ben-Moshe, Shalev Itzkovitz

**Affiliations:** Department of Molecular Cell Biology, Weizmann Institute of Science, Rehovot, Israel; Department of Molecular Cell Biology, Weizmann Institute of Science, Rehovot, Israelshalev.itzkovitz@weizmann.ac.il

**Keywords:** gene expression, single cell, single molecule, fluorescence in situ, mathematical modeling, theory, Mouse

## Abstract

How are cells able to maintain constant levels of mRNA when the number of genes in a cell doubles ahead of cell division?

**Related research article** Skinner SO, Xu H, Nagarkar-Jaiswal S, Freire PR, Zwaka TP, Golding I. 2016. Single-cell analysis of transcription kinetics across the cell cycle. *eLife* **5**:e12175. doi: 10.7554/eLife.12175

**Image** Mouse embryonic stem cells compensate for gene duplication by switching each copy of the gene to an open state less often

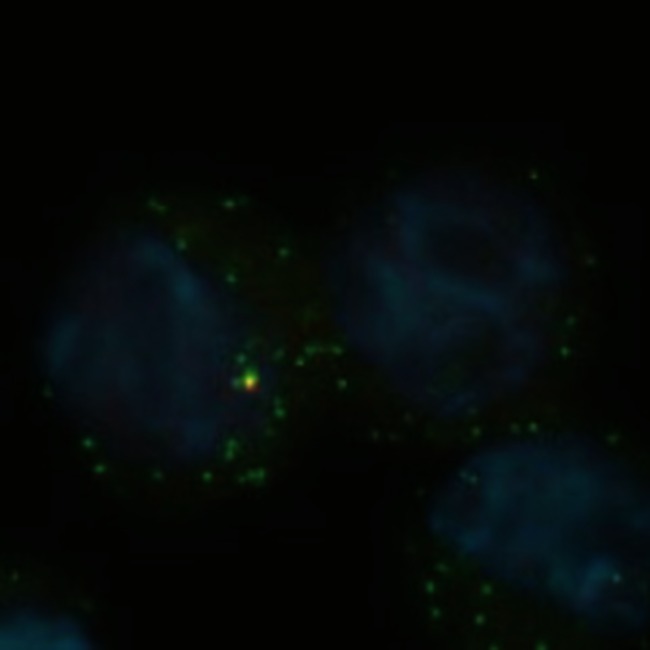

Imagine trying to maintain a constant speed while driving a car. Sounds simple enough, but what if you’re only allowed to press the accelerator pedal intermittently. As strange as this driving technique seems, cells often behave in a comparable manner when transcribing genes to produce messenger RNA (mRNA) molecules. The mRNA molecules are produced when a region of the gene called the promoter is open: since these promoters randomly switch between open and closed states, the mRNA molecules are produced in bursts. Cells also degrade mRNA, just as friction from the road reduces the speed of a car. As a result, mRNA levels will rise and fall over time (Raj and van Oudenaarden, 2008).

Now let’s go back to our car analogy and add another complication. What if a second driver miraculously appears from time to time, controlling their accelerator independently of yours? How could you possibly avoid breaking the speed limit now? With double the acceleration you would have to compensate by either reducing how often or how hard you pressed the accelerator pedal.

Cells face a similar challenge when they replicate (Figure 1). During some phases of the cell cycle, the cell contains twice as many copies of each gene as usual, which could double the amount of mRNA that is produced. How do replicating cells compensate for this so that mRNA levels remain constant? Now, in eLife, Ido Golding and colleagues – including Samuel Skinner as first author – present an elegant framework that can be used to address this question (Skinner et al., 2016).

**Figure 1.**
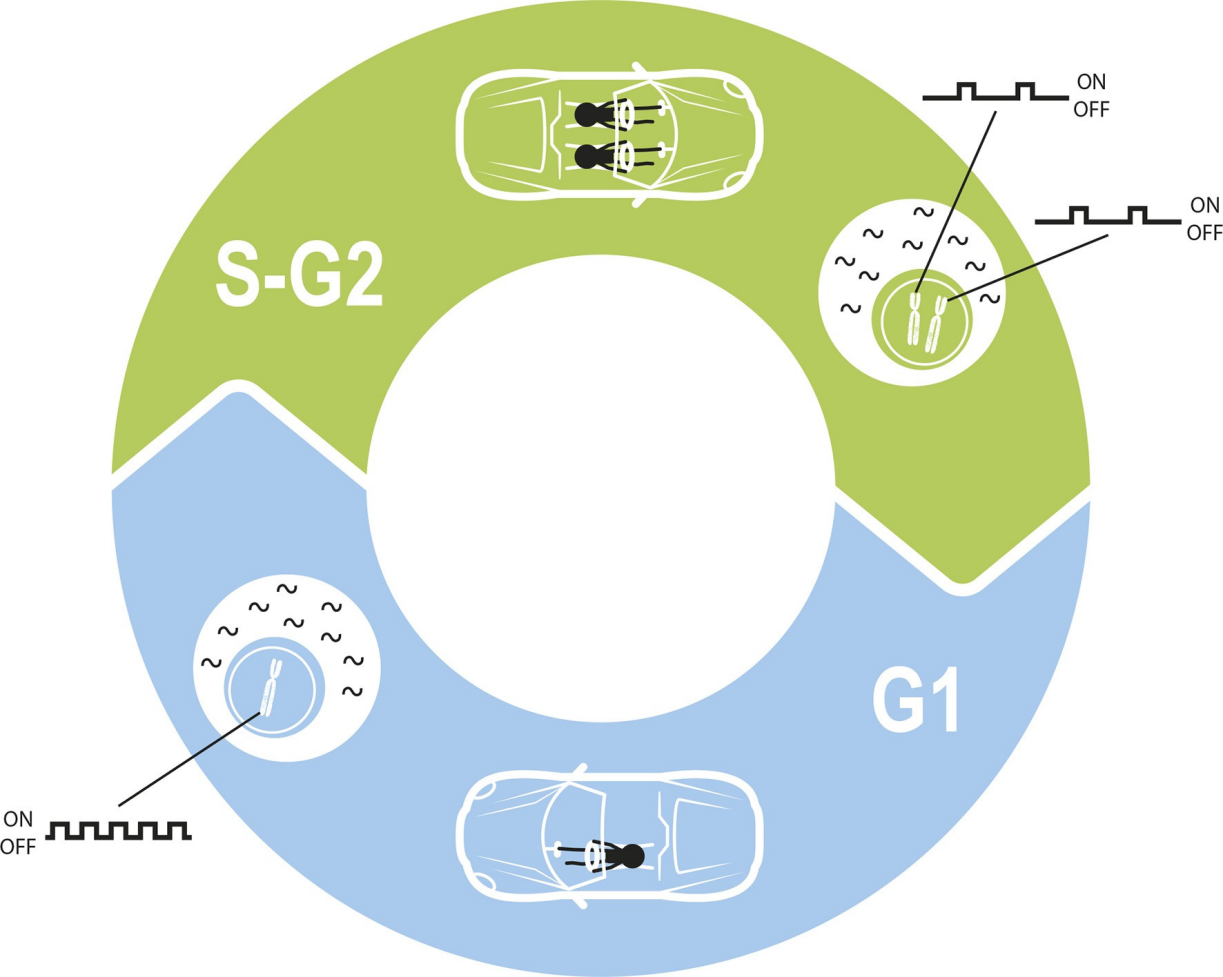
Maintaining constant levels of mRNA throughout the cell cycle. During the G1 phase of the cell cycle (blue), the promoter for a given gene opens and closes to produce mRNA molecules (black waves) in bursts. However, during the S phase and G2 phase of the cell cycle (green), the cell contains twice as many copies of each gene as a result of replication. Cells must therefore compensate for these extra copies in order to maintain constant mRNA levels throughout the cell cycle. Skinner et al. found that this compensation is achieved by reducing the number of times per hour that the promoter is open. The challenge of maintaining a constant level of mRNA is similar to the challenge of maintaining a constant speed in a car in which a second driver periodically appears.

Using a technique called single-molecule mRNA fluorescence in-situ hybridization, Skinner et al. – who are based at Baylor College of Medicine, Rice University, the University of Illinois at Urbana-Champaign and the Icahn School of Medicine at Mount Sinai – quantified the levels of mature and newly transcribed mRNA molecules in individual mouse embryonic stem cells. They also measured how much DNA each cell contained and used this to work out which phase of the cell cycle the cell was in. These measurements allowed the activity of each copy of a particular gene to be analyzed before and after gene replication.

Skinner et al. found that two genes that are important in embryonic stem cells, *Nanog* and *Oct4,* are transcribed in a “bursty” manner. Furthermore, this activity reduces after DNA replication to compensate for the extra DNA in the cell, thus equalizing mRNA levels over the cell cycle. A similar "dosage compensation" mechanism for altering the rate of transcription following DNA replication has been documented in other biological systems (Fraser and Nurse, 1978; Padovan-Merhar et al., 2015; Voichek et al., 2016; Yunger et al., 2010).

There are three parameters that could be reduced to achieve dosage compensation: how often a given promoter opens per hour (the burst frequency); how long it remains open for; and the rate at which mRNA is produced from an open promoter (burst size). To identify which of these parameters are relevant in mouse stem cells, Skinner et al. developed a model that accounts for the fact that the number of copies of a gene changes during the cell cycle.

A simplified model in which the burst frequency was the only parameter that changed during the cell cycle resulted in an excellent fit to the experimental data. This means that cells seem to compensate for gene replication by having each copy switch to an open state less often, rather than by reducing how many mRNAs they produce when open. Skinner et al. also found that the burst frequency was the parameter that differed most between their two studied genes, *Oct4* and *Nanog*. These results reinforce the findings of other recent studies in mammalian cells, which identified the importance of regulating transcription rate through changes in burst frequency. This regulation can occur throughout the cell cycle (Padovan-Merhar et al., 2015), in response to transcription factor levels (Senecal et al., 2014) or in liver genes in response to metabolic stimuli (Bahar Halpern et al., 2015).

The cell cycle poses additional challenges for cells. For one, cell division immediately halves mRNA production, and so mRNA levels must increase in preparation for this event. This is achieved through a mechanism that monitors the volume of the cell and increases burst size accordingly (Padovan-Merhar et al., 2015). In addition, mRNA degradation rates vary greatly between genes, posing different constraints on burst parameters and their compensation mechanisms. Again there are parallels with driving: it is much more difficult to maintain a constant speed on a dirt track than on a major highway.

Finally, there are cases where cells amplify variability in mRNA levels to generate differences between cells. An example is the bacterial stress response, where variable mRNA levels of key genes can help to vary the response (Eldar and Elowitz, 2010). Stem cell differentiation strategies may also change depending on the stage of the cell cycle that has been reached (Pauklin and Vallier, 2013). Skinner et al. now provide the tools to identify the burst parameters that vary throughout the cell cycle for any gene and biological system of interest.
